# Analysis of human invasive cytotrophoblasts demonstrates mosaic aneuploidy

**DOI:** 10.1371/journal.pone.0284317

**Published:** 2023-07-21

**Authors:** Jingly F. Weier, Christy Ferlatte, Adolf Baumgartner, Ha Nam Nguyen, Beatrice A. Weier, Heinz-Ulrich G. Weier

**Affiliations:** 1 Department of Obstetrics, Gynecology, and Reproductive Sciences, University of California (U.C.), San Francisco, California, United States of America; 2 Life Sciences Division, U.C. E.O. Lawrence Berkeley National Laboratory, Berkeley, California, United States of America; 3 Golden State Dermatology, Walnut Creek, California, United States of America; Virginia Tech, UNITED STATES

## Abstract

A total of 24 chromosome-specific fluorescence *in situ* hybridization probes for interphase nucleus analysis were developed to determine the chromosomal content of individual human invasive cytotrophoblasts derived from *in* v*itro* cultured assays. At least 75% of invasive cytotrophoblasts were hyperdiploid and the total number of chromosomes ranged from 47 to 61. The results also demonstrated that these hyperdiploid invasive cytotrophoblasts showed significant heterogeneity. The most copy number gains were observed for chromosomes 13, 14, 15, 19, 21, and 22 with average copy number greater than 2.3. A parallel study using primary invasive cytotrophoblasts also showed a similar trend of copy number changes. Conclusively, 24-chromosome analysis of human non-proliferating cytotrophoblasts (interphase nuclei) was achieved. Hyperdiploidy and chromosomal heterogeneity without endoduplication in invasive cytotrophoblasts may suggest a selective advantage for invasion and short lifespan during normal placental development.

## Introduction

The placenta is a transient organ in mammalian pregnancy. It functions as a fetomaternal organ which develops from the trophectoderm of the blastocyst after uterine implantation, separating it from the inner cell mass that will later form the embryo. During the early placentation phase, extravillous trophoblasts are part of a highly invasive tumor-like structure that ensures effective implantation in a short period of time [1, 2]. Cytotrophoblasts (CTBs), stem cells of trophoblasts, can differentiate into either villous CTBs or extravillous CTBs. While fusion of villous CTBs yields syncytiotrophoblasts (STBs), extravillous CTBs differentiate into invasive CTBs [3–5]. These invading CTBs, which migrate from anchoring villi, are commonly termed extravillous trophoblasts. Studies have shown that extravillous trophoblasts enter the maternal circulatory system during the first trimester of pregnancy. Recently, extravillous trophoblasts have been clinically used for noninvasive prenatal testing (NIPT) to screen for fetal aneuploidy and for chromosomal duplications and deletions [6–8]. Therefore, understanding the chromosomal status of these extravillous trophoblasts becomes an important issue for placental biology and prenatal screening.

Genetic screening using comparative genomic hybridization and next-generation sequencing on trophectodermal cells biopsied from blastocysts at days 5–6 showed aneuploidy rates of 56% and 41%, respectively [9, 10]. Chorionic villus sampling (CVS) at 10 to 12 gestational weeks, only examining proliferating cells from placental villi, showed a 2% aneuploidy rate [11, 12]. To date, many genetic studies have only examined the trophectoderm of blastocysts, floating villi, and the cells these structures contain, including the mesenchyme. Cytogenetic studies on invasive extravillous trophoblasts are still limited due to these cells exhibiting permanent cell cycle withdrawal [13]. Endoduplication with elevated ploidy level has been proposed for invasive CTBs [14, 15]. One study showed that extravillous trophoblasts obtained from the cervix did undergo endoreduplication to form ploidy levels of 4N or 8N which had been confirmed by fluorescence *in situ* hybridization (FISH) targeting chromosomes X,Y, and 21 [16]. Our previous study [17] did not observe endoduplication in invasive CTBs but showed that more than 50% of invasive CTBs in uncomplicated pregnancies were chromosomally abnormal for chromosomes X, Y, and 16. We further showed that male invasive CTBs have higher rates of aneuploidy and hyperdiploidy than female invasive CTBs. When 12 different chromosomes (X, Y, 3, 6, 8, 9, 10, 11, 12, 16, 17, and 18) were targeted to evaluate invasive CTBs, the aneuploidy rate was very high as 97.3% [17].

To completely map the aneuploidy status of invasive CTBs, we developed a chromosome-specific probe for all 24 human chromosomes (1–22, X, and Y). In this most comprehensive study to date, we have successfully used 24 unique chromosome-specific probes [18] to fully enumerate the chromosomal content of individual invasive CTBs. By determining the chromosome-specific frequencies of aneuploidy and mosaicism in CTBs, we provide valuable insights into the underlying biological mechanisms of the CTB invasion process.

## Materials and methods

### Placental tissues and isolated invasive CTBs cultured *in vitro*

All procedures followed protocols approved by the UCSF and Lawrence Berkeley National Laboratory Committees on Human Research regarding use of discarded human placenta for research. Portions of the placenta and basal plate (maternal-fetal interface) were collected immediately after elective pregnancy terminations for non-medical reasons during the first or second trimester. The gestational age ranged from 5–7 weeks and 15–23 weeks for first- and second-trimester placentas, respectively.

**Fig 1:**
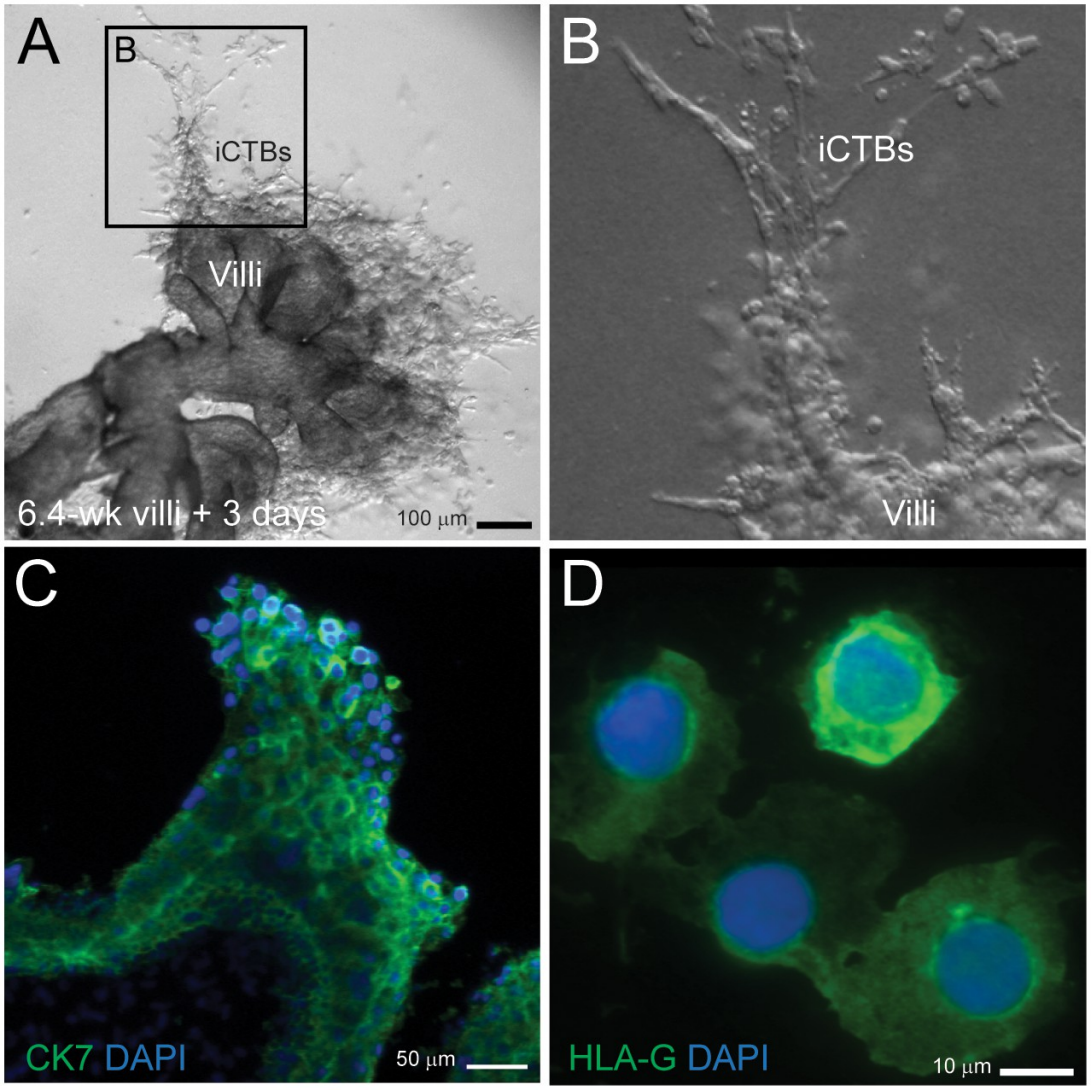
First trimester placental villi grown on Matrigel to obtain invasive cytotrophoblasts (iCTBs) *in vitro*. (A, B) Representative images of iCTBs outgrowth from a 6.4wk placental villi culture explants for 3 days. (C) Immunostaining of first trimester placenta villi culture explant showed most iCTBs are positive for Cytokeratin-7 (CK-7). (D) CTBs on the tip of the villi differentiated into iCTBs were harvested and fixed on glass slides showing positive for HLA-G.

To obtain invasive CTBs *in vitro*, placental villi from the first trimester were grown on Matrigel [4]. Before being placed in a sterile 10-cm Petri dish, primary first trimester (5–7 weeks) villi were washed in cytowash (Dulbecco’s Modified Eagle Medium /H21 + 1% glutamine + 2.5% FBS, + 50 μg/ml gentamycin + 1% Penicillin/Streptomycin, UCSF Cell Culture Facility) several times to remove dead cells and blood cells. Under a dissection microscope, tips of anchoring villi were cut, isolated from the placenta, and placed onto Millicell-cm well plate inserts (Millipore, Billerica, MA) pre-coated with 100% Matrigel (BD Biosciences, San Jose, CA). The villi were cultured in low level medium (Dulbecco’s Modified Eagle Medium / F12, 20% fetal bovine serum (FBS) and 1% antibiotic/antimycotic mixture, UCSF Cell Culture Facility) to prevent the villi from floating off. After three days of culture at 37°C and 5% CO_2_, the villi were dissected and removed from the plate leaving behind invasive CTBs embedded within the Matrigel layer (Fig 1A and 1B). The invasive CTBs were further isolated by enzymatic digestion with dispase and collagenase (1 mg/ml each, Invitrogen, Carlsbad, CA). Isolated invasive CTBs were spun onto microscope slides (Thermo Shandon, Pittsburg, PA) by a Cytospin^®^ centrifuge, fixed in methanol at 4°C for 10 minutes and stored at -20°C. The purity of invasive CTBs was determined using immunostaining with Cytokeratin-7, HLA-G described in previous studies [17, 19] using mouse anti-human Cytokeratin-7 (diluted 1:50, vol/vol, Dako/Agilent, USA, cat # M701829-2), and mouse monoclonal HLA-G (clone 4H84, diluted 1:50, vol/vol, a gift from Dr. S. Fisher, UCSF).

Second trimester placenta tissues were used for the *in-vivo* study. Areas with floating villi and portions of anchoring villi together with their uterine attachment sites were biopsied. All samples were washed in ice-cold phosphate-buffered saline (PBS), then fixed with freshly prepared 3% paraformaldehyde (PFA) in PBS for 30 min at 4°C. After two wash steps in PBS at 20°C, tissues were immersed in a sucrose series (5%, 10%, 15% in PBS; 15 min/step at 4°C). Finally, the samples were incubated for 20 min at 4°C in a 1:1 (vol/vol) mixture of 15% sucrose in PBS and “optimum cutting temperature” formulation of water-soluble sucrose and resins (Sakura Tissue-Tek O.C.T. compound), embedded in O.C.T. compound, frozen in liquid nitrogen and stored at -80°C [20]. Placental tissue sections (5-8 μm thick) were cut using a cryostat (Slee International Inc., Tiverton, RI), collected on precleaned ProbeOn Plus microscope slides (Thermo Fisher Scientific, Santa Clara, CA) and stored at -20°C.

### Fluorescence *in situ* hybridization

Table 1 lists the fluorochrome labeling scheme for all 24 chromosome-specific DNA probes. The details of probe preparation and labeling were previously described by Baumgartner and colleagues [18]. Four sets of probes were prepared in-house, and each set included six chromosome-specific DNA probes labeled with six different fluorochromes (DEAC, Spectrum Green, Spectrum Orange, Spectrum Red, Cy5, and Cy5.5). For each set, 10 μl of hybridization mixture was prepared by adding 2~6 μl of each DNA probe, 6~15 μl of Human Cot 1 DNA (1 mg/ml, Invitrogen), 2 μl of Salmon Sperm DNA (10 mg/ml, Invitrogen, Carlsbad, CA), 1 μl glycogen (5 mg/ml, Thermo Fisher Scientific, Santa Clara, CA) and 50~85 μl of ethanol. The mixture was then incubated at -20°C for 90 min before being centrifuged. The DNA-probe pellet was air-dried, resuspended in 3 μl H_2_O and 7 μl of LSI/WCP Hybridization Buffer (Abbott Molecular, Des Plaines, IL).

**Table 1:** Fluorochrome-labeling scheme for 24 chromosome-specific DNA probes.

Fluorochrome	Set I Chr.	Set II Chr.	Set III Chr.	Set IV Chr.
DEAC	14	Y	5	8
Spectrum Green^®^	21	19	3	6
Spectrum Orange^®^	22	15	4	10
Spectrum Red^®^	20	17	2	7
Cy5	13	X	12	11
Cy5.5	16	18	9*	1

*The chromosome 9-Cy5.5 probe developed in-house showed dim hybridization signals in the set III mixture. The slides were re-hybridized with a single centromere-specific chromosome 9-Spectrum Orange probe to fully karyotype the cells.

Slides with isolated invasive CTBs and normal lymphocytes on commercially available control slides (ProbeChek: 0% trisomy 8/12, Abbott Molecular, Des Plaines, IL) were incubated in Carnoy’s fixative (3 parts methanol plus 1 part acetic acid) for 10 min followed by immersion in 2× SSC (0.3 M NaCl, 0.03 M Na_3_citrate·2 H_2_O, pH 7.0) for 1 h at 37°C. Slides were then dehydrated in an ethanol series (70%, 80%, and 100% ethanol for 2 min each) and air-dried.

Slides with fixed isolated cells were denatured for 4 min at 76°C in 70% formamide (FA) / 2× SSC, pH 7.0, and then dehydrated in 70%, 80%, and 100% ethanol (2 min per step) before being air-dried. The hybridization probe mixture was denatured for 10 min at 76°C followed by an incubation at 37°C for 30 min. Ten microliters of denatured hybridization probe mixture were then applied to each slide, coverslips were added and sealed with rubber cement. The hybridization reaction was allowed to proceed at 37°C for 40 h. Following hybridization, the coverslips were removed by immersing the slides in 2× SSC. The slides were transferred in 0.1× SSC for 2 min at 43°C then twice in 2× SSC (10 min each time, 22°C). Glass coverslips were mounted onto the slides with 8 μl of 4′,6-diamidino-2-phenylindole (DAPI, 0.5 μg/ml, Calbiochem, La Jolla, CA) dissolved in anti-fade medium [21]. For repeated hybridizations, the coverslips were rinsed off the slides followed by a wash in water before being dehydrated again in an ethanol series. Slides were only denatured for 2 min at 76°C in FA / 2× SSC solution for any subsequent hybridization at 37°C for 40 h. The first set (set I) of six chromosome-specific probes (chromosomes 13, 14, 16, 20, 21, and 22) were analyzed in interphase nuclei (male lymphocytes and invasive CTBs). Then, after a washing step and repeated hybridization with set II (chromosomes X, Y, 15, 17, 18, and 19) was analyzed followed by set III (chromosome 2, 3, 4, 5, 9, and 12), and set IV (chromosomes 1, 6, 7, 8, 10, and 11).

FISH on tissue sections were previously described in detail by Weier and colleagues [17]. Briefly, tissues adherent to slides were incubated in Carnoy’s fixative for 5 min at 20°C, and then placed on a hot plate at 45°C for 5 min before pepsin pretreatment (50-100 μg/ml pepsin (Amersco, Solon, OH) in 0.01 N HCl) for 10~20 min at 37°C, post-fixed in 4% PFA in PBS and then dehydrated using an alcohol series. Tissues on slides and FISH probes were co-denatured on a hot plate at 85°C for 10 min, followed by hybridization at 37°C for 40 h. Slides were placed in a wash solution and FISH signals were scored under the microscope. FISH was carried out evaluating thirteen chromosomes (6, 7, 8, 9, 13, 14, 15, 16, 18, 19, 21, 22, and X) on normal second trimester placental tissue sections (5~8 μm). Each section was hybridized with two to three chromosome-specific individual probes.

### Image acquisition and analysis

Fluorescence microscopy was performed on a Zeiss Axioskop microscope equipped with a SKY filter set (ChromaTechnology, Brattleboro, VT) suitable for Spectral Imaging for simultaneous observation of the fluorochromes, and with a DAPI filter (ChromaTechnology, Brattleboro, VT) for the detection of the counterstain. Images were collected using a cooled CCD camera (CCD-1300DS, VDS Vosskuehler, Osnabrück, Germany) [22]. The Spectral imaging filter allowed excitation and simultaneous observation of fluorescence from DAPI, Texas Red / rhodamine, DEAC / Spectrum Aqua, FITC, CY5, and Cy5.5 (ChromaTechnology, Brattleboro, VT). The excitation spectrums of these six different fluorochromes were easily separated by the spectrum Imaging system [22–24]. The spectral information was displayed by assigned RGB colors to three areas of interest in the spectrum. Based on the measured spectrum of each signal domain, a classified color image was generated. For repeated hybridizations, cells were localized by recording the coordinates on the microscope. Further processing and printing of the images were done using the image processing software Adobe Photoshop (Adobe Systems Inc., San Jose, CA).

For scoring, hybridization signals were counted according to the criteria published by Hopman and colleagues [25]: pairs of fluorescence signals that were spaced less than the diameter of a signal domain apart were counted as one chromosome, and pairs of signals that were farther apart than a signal domain were counted as two chromosomes. A total of 100 normal male lymphocytes and 600 invasive CTBs from five different Matrigel cultured villi were analyzed. Among these cells, a total of 329 individual invasive CTBs from four cultured villi were recorded and analyzed for all 24 chromosomes. For each tissue section, 40 individual cells were analyzed from three different areas in which single cells were resolved (invasive CTBs in basal plate, syncytiotrophoblasts in floating villi, and mesenchyme in villus core). Chromosome-specific aneuploidy included hypodiploidy and hyperdiploidy (compared to the diploid set, fewer or additional chromosomes are present in the nucleus).

### Statistical analysis

**Fig 2:**
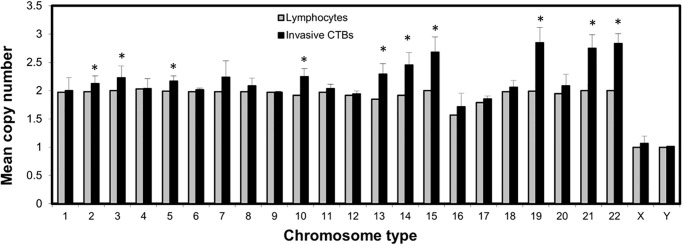
The mean copy number of each chromosome for normal male lymphocytes and invasive cytotrophoblasts derived from *in-vitro* Matrigel culture. Invasive CTBs with significant gain of extra copies of chromosomes 2, 3, 5, 10, 13, 14, 15, 19, 21 and 22. (*) (*P* < 0.05). Samples contained a total of 100 lymphocytes and 600 invasive CTBs from two female and three male placentas.

For statistical analysis, Rstudio (version 2022.12.0) was used. Using this software, Fig 4, was created with the ggplot2 package available in Rstudio. The analyses performed were one-sample t-tests, and two sample t-tests. Random sampling, independence, and normality were assumed due to the random nature of the cells selected. An alpha threshold of 0.05 was used for Figs 2 and 7. When multiple comparisons were done against the same set of data the alpha threshold was lowered; for Fig 6 BP data was compared to both VC and ST data, therefore the alpha threshold was 0.025. T-scores were calculated using means and standard deviations, which then were converted to p-values using the pt() function in Rstudio. Degrees of freedom varied based on the amount of data given per each point.

## Results

We developed a 3-dimensional culture system to generate invasive CTBs from placenta villi (Fig 1A and 1B). As predicted, CTBs from villi tips developed an invasive phenotype in culture. The tip of the villi showed the presence of human cytokeratin-7 (Fig 1C). Invasive CTBs from the employed *in-vitro* assay were all positive for HLA-G (Fig 1D) indicating that the dissected CTBs from the tip of the villi after 72-hr culture *in vitro* were similar to primary invasive CTBs. A total of five samples (three male and two female) from *in-vitro* Matrigel cultures were separately collected for cytogenetic analysis. Four different sets of FISH probes were used for successive hybridizations to cover 24 human chromosomes (Table 1: Fluorochrome-labeling scheme for 24 chromosome-specific DNA probes).

All FISH signals in metaphases and interphases from normal male control slides were unambiguous, strong, and without cross-hybridization to other chromosomes [18]. The exception was chromosome 9 in set III which did produce a strong signal when tested by itself but was rather dim when co-hybridized with the rest of set III probes. One hundred normal male lymphocyte nuclei were hybridized with the four FISH probe sets and analyzed as controls. Fig 2 shows the mean copy number of each chromosome-specific probe in male lymphocyte nuclei with most of the autosomes showing two copies each, except for partial one-copy loss of chromosomes 16 and 17. The mean copy number for chromosome 16 and 17 was 1.57 and 1.79, while others ranged from 1.85 (chromosome 13) to 2.03 (chromosome 4). The male lymphocytes FISH results demonstrated that the chromosome-specific probes developed in-house, Spectral Imaging analysis on single interphase nucleus, and repeated hybridizations with four sets of probes were suitable to study the chromosomal composition of invasive CTBs.

**Fig 3:**
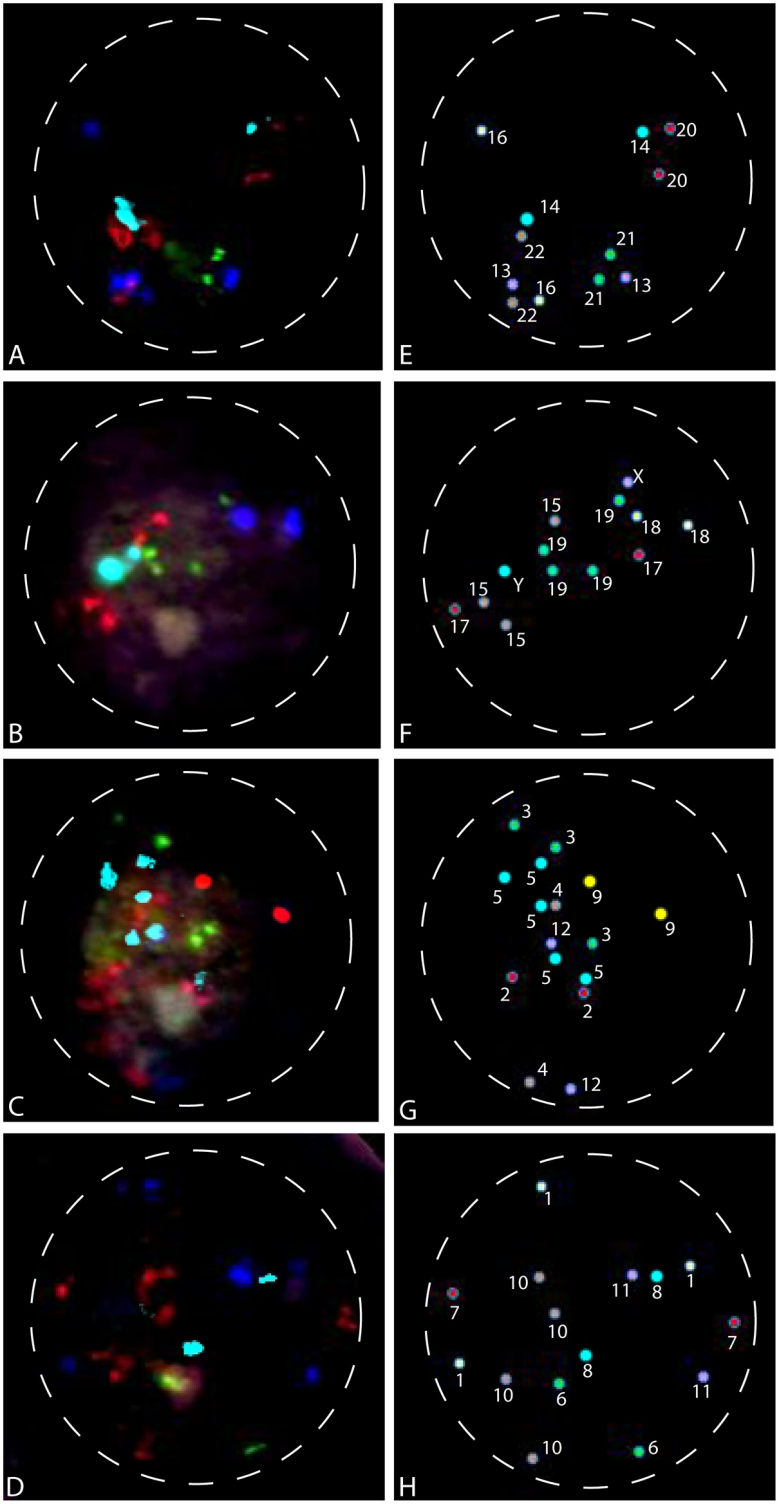
Analysis of 24 chromosomes on one invasive cytotrophoblast using fluorescence *in situ* hybridization. (A-D) FISH probe sets I to IV were hybridized on the same invasive CTB by sequentially repeated hybridizations. (E-F) The corresponding classified image from Spectral Imaging system (pseudo-colors) revealed the following hyperdiploid karyotype for this CTB: nuc ish 56,XY,+1,+3,+5,+5,+5,+10,+10,+15,+19,+19.

A total of 329 invasive CTBs (iCTBs-1, 98 male cells; iCTBs-2, 91 male cells; iCTBs-3, 57 female cells; iCTBs-4, 83 male cells) were analyzed for all 24 chromosomes. Fig 3 shows an example of FISH results for one invasive CTB by repeated hybridizations with probe set I (Fig 3A), probe set II (Fig 3B), probe set III (Fig 3C), and probe set IV (Fig 3D). Fig 3A–3D show the RGB FISH images while Fig 3E–3H display the corresponding classified image from Spectral Imaging system (pseudo-colors). Fig 3E shows an invasive CTB with two copies of chromosomes 13, 14, 16, 20, 21, and 22. Fig 3F identifies this invasive CTB as a male cell with two copies of chromosomes 17, 18, three copies of chromosome 15, and four copies of chromosome 19. Fig 3G shows the same cell having two copies of chromosomes 2, 4, 9, 12, three copies of chromosome 3, and five copies of chromosome 5. Please note that one signal on the left was not counted as one chromosome 5. This signal was a background artifact with a spectrum not in our designed fluorochrome listed in Table 1. Fig 3H shows that this cell has two copies of chromosomes 6, 7, 8, 11, three copies of chromosome 1, and four copies of chromosome 10. In summary, this was a hyperdiploid male invasive CTB with 56 chromosomes, containing extra copies of chromosomes 1, 3, 5, 10, 15, and 19 (nuc ish 56,XY,+1,+3,+5,+5,+5,+10,+10,+15,+19,+19).

**Fig 4:**
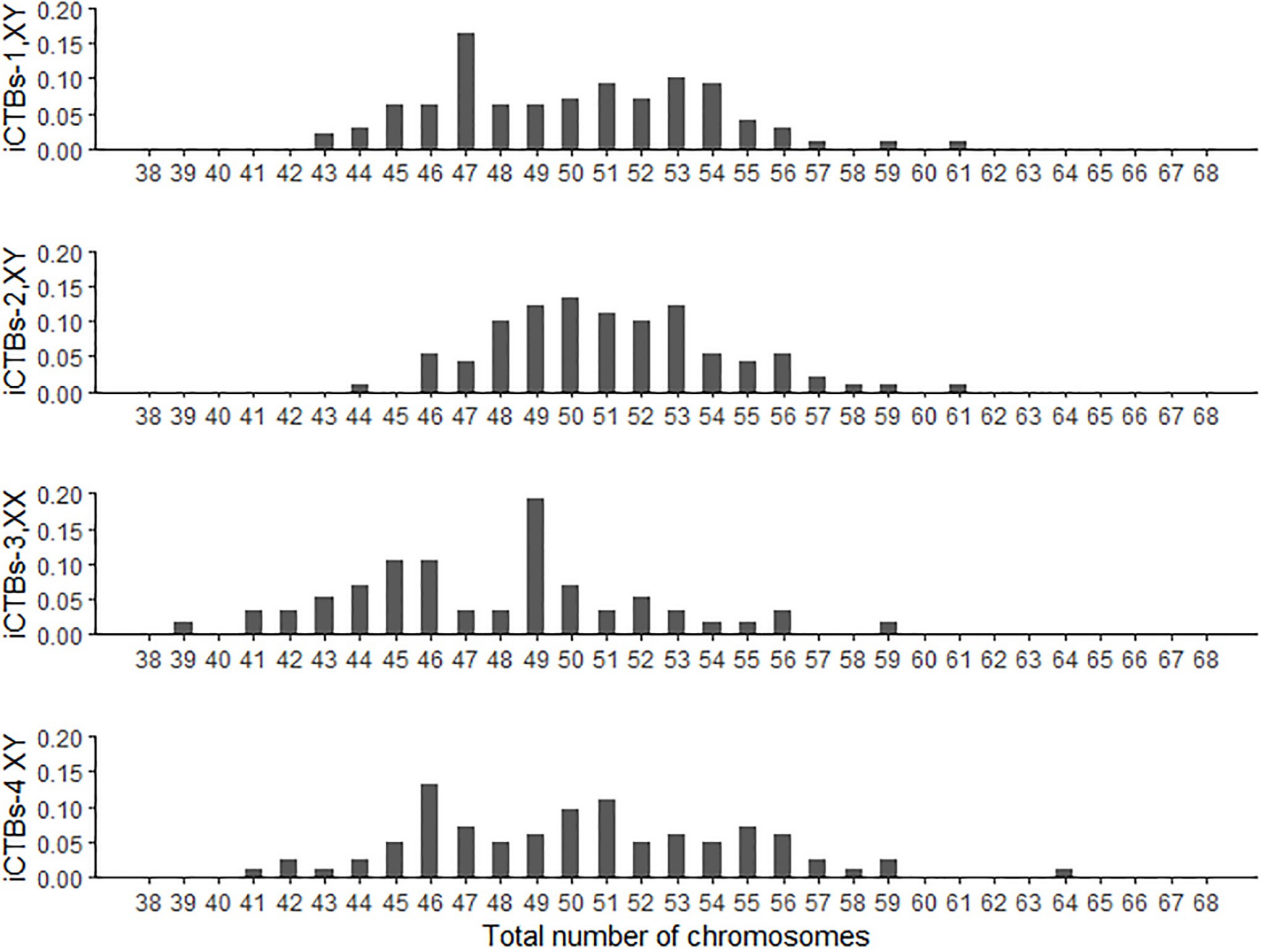
Total number of chromosomes for four different invasive cytotrophoblasts. At least 75% of the male cells and 54% of the female cells were hyperdiploid (Total number of chromosomes > 46). Male iCTBs-1: 98 cells; Male iCTBs-2: 91 cells; Female iCTBs-3: 57 cells; Male iCTBs-4: 83 cells). Y-axes represent the Fraction of cells.

Fig 4 shows the proportion of cells for four different samples, with the total number of chromosomes ranging from 38 to 68. The most common total number of chromosomes for iCTBs-1 sample were 47 (16.3%), 51 (9.2%), 53 (10.2%), and 54 (9.2%). For iCTBs-2 sample, the most common total number of chromosomes were 49 (12.1%), 50 (13.2%), 51 (11%), and 53 (12.1%). For iCTBs-3 sample, the most common total number of chromosomes were 49 (19.3%), 45 (10.5%) and 46 (10.5%). Finally, for iCTBs-4 sample, the most common total number of chromosomes were 46 (13.3%), 50 (9.6%), and 51 (10.8%). Overall, more than 75% of the male invasive CTBs (iCTBs-1, 82.7%; iCTBs-2, 93.4%; iCTBs-4, 74.7%), and 54.4% of the female invasive CTBs (iCTB-3) were hyperdiploid. This is consistent with our previous study showing the rate of hyperdiploid cells in male invasive CTBs is higher than in female invasive CTBs.

**Fig 5:**
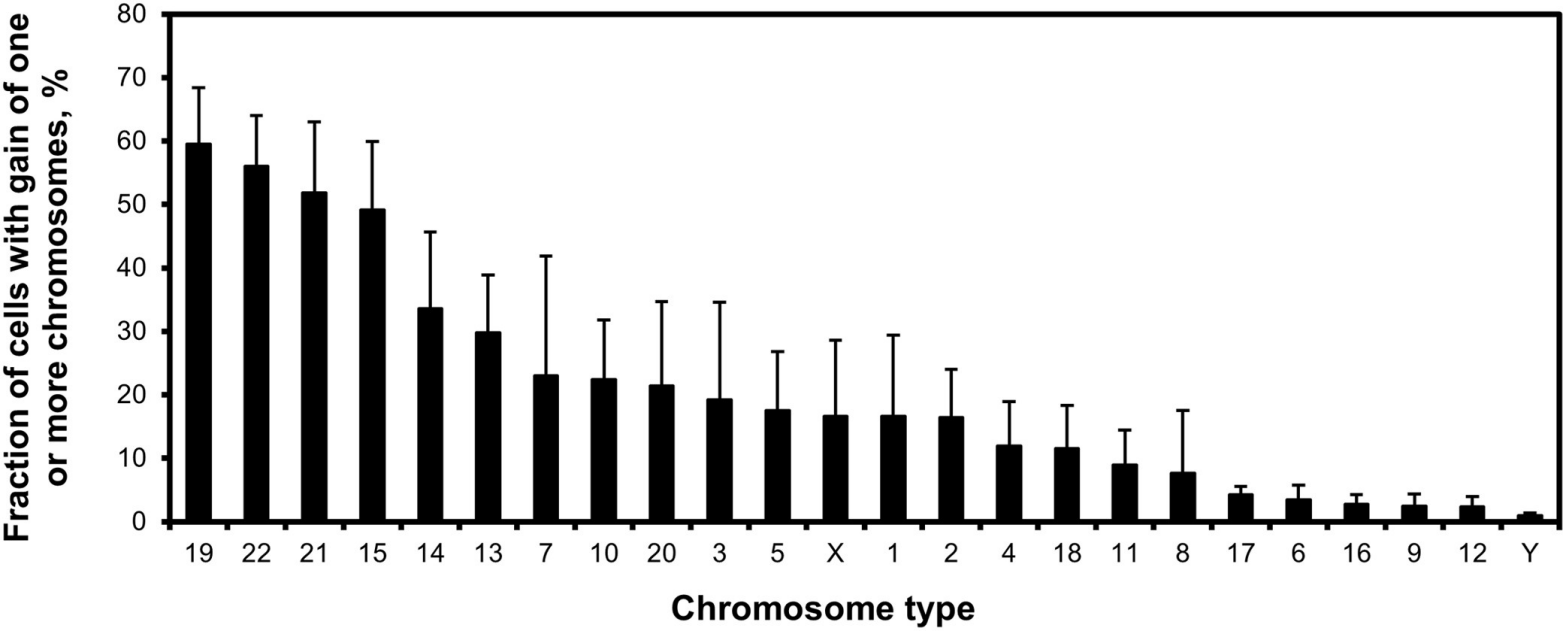
Hyperploid rate of each chromosome on invasive cytotrophoblasts. The most common chromosomal gains affected chromosomes 13, 14, 15, 19, 21, and 22. Samples contain 600 invasive CTBs from two female and three male placentas.

To evaluate which chromosome had the most gains in invasive CTBs, the mean copy number of each chromosome (chromosomes 1–22, X and X) were analyzed and shown in Fig 2, which also shows the comparison with normal male lymphocytes. There were gains of chromosomes 2 (2p16.1–15), 3 (3q27.3), 5 (5q23.1), 10 (10cen, α-sat), 13 (13q21.31), 14 (14q13.3), 15 (15q25.3), 19 (19q13.2), 21 (21q22), and 22 (22q11)–with parentheses indicating the FISH-probes’ target regions [18]. The mean copy number ranged from 2.12 for chromosome 2 to 2.85 for chromosome 19. One sample t-tests were performed (alpha = 0.05) comparing the mean lymphocyte to the invasive CTBs data. Significant differences were found on chromosomes 2, 3, 5, 10, 13, 14, 15, 19, 21, and 22. The occasional loss of chromosomes 16 (16qh, sat II) and 17 (17cen, α-sat) was also observed in invasive CTBs. Fig 5 shows the fraction of cells with a gain of one or more copies of each chromosome. Extra copies of chromosome 19 in invasive CTBs were the most common, with 59.5% of the invasive CTBs having extra copies. The next most common gains were for chromosomes 22 (56%), 21 (52%), 15 (49%), 14 (34%), and 13 (30%). The least common gains were chromosomes 6, 9, 12, and 16. Conclusively, invasive CTBs were hyperploidy with the total number of chromosomes ranging from 47 to 61 and with most gains on chromosomes 13, 14, 15, 19, 21 and 22.

**Fig 6:**
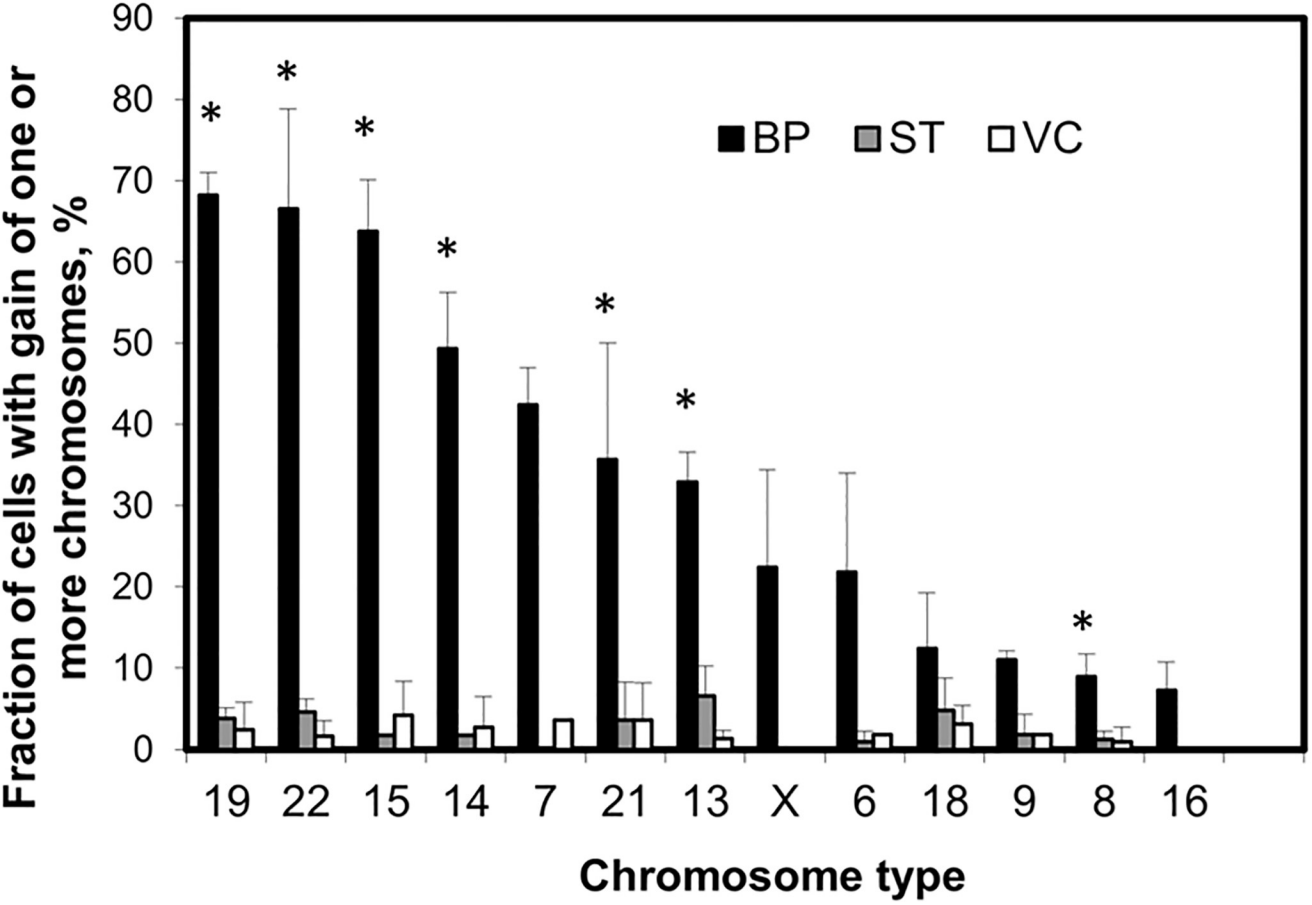
Fluorescence *in situ* hybridization analysis of second trimester placental tissue sections. Thirteen different chromosome probes were hybridized on tissue sections showed that hyperdiploidy was found more often in cells of the uterine wall (BP, invasive cells in basal plate) than in those of the floating villi (ST: syncytiotrophoblasts; VC: mesenchyme in villus core). Significant differences between the means were found on chromosomes 19, 22, 15, 14, 21, 13, and 8. (*) (*P* < 0.025). Each chromosome was tested on 2–6 different second trimester placentas.

**Fig 7:**
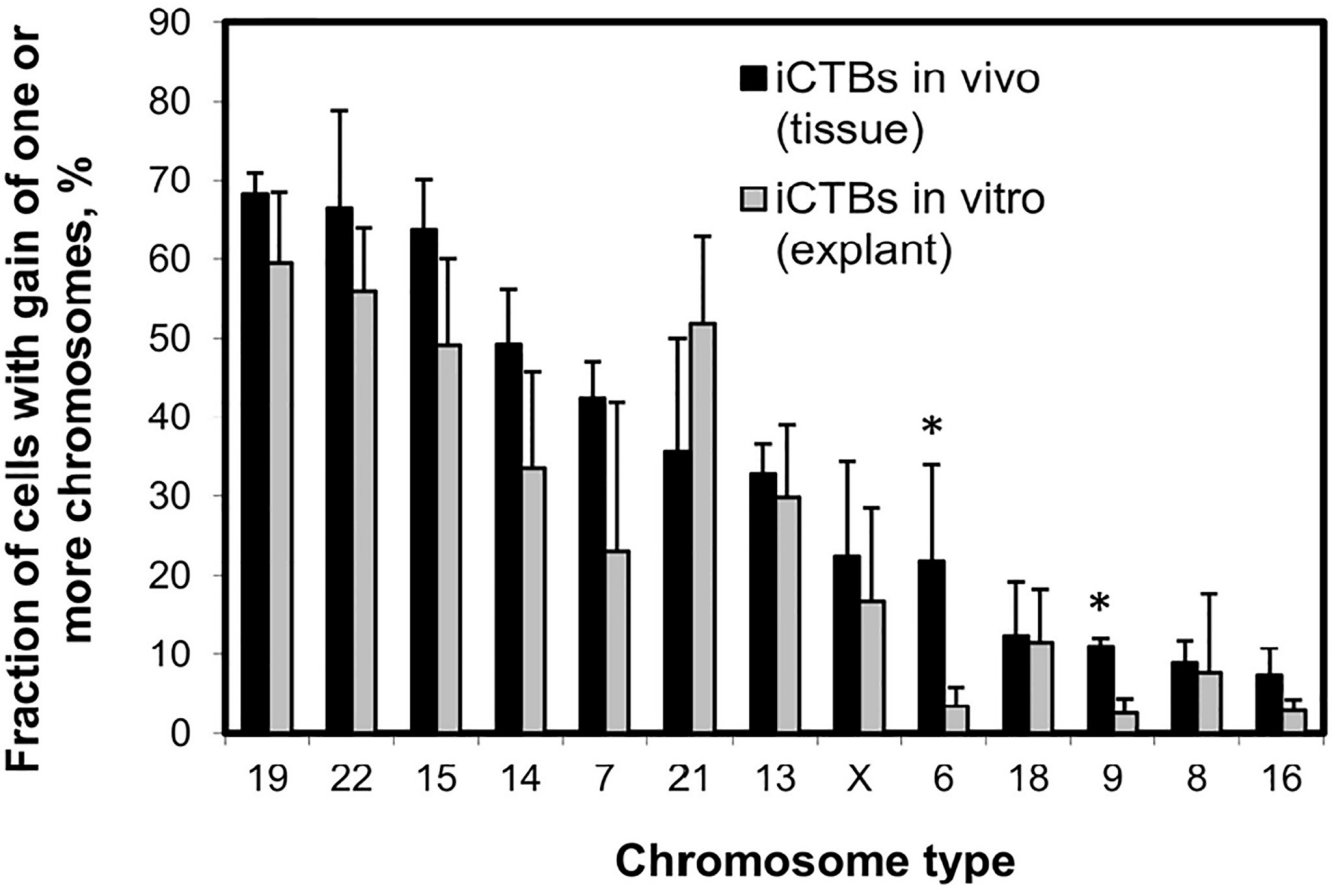
The hyperdiploidy rate on different chromosomes for invasive CTBs *in vivo* and *in vitro*. The hyperdiploidy rate of different chromosomes in invasive CTBs correlated with *in vivo* tissues (2–6 samples) and *in vitro* studies (5 samples). The most common chromosomal gains were found in chromosomes 15, 19, and 22. Significant differences between the means were found on chromosomes 6 and 9. (*) (*P* < 0.05).

To determine where similar chromosomal gains occurred *in vivo*, tissue sections from second trimester placenta were analyzed with thirteen different chromosomes (chromosomes 6, 7, 8, 9, 13, 14, 15, 16, 18, 19, 21, 22, and X). The chosen chromosomes were based on the most and least common copy number changes in isolated invasive CTBs derived from the *in-vitro* extraction. FISH signals of each chromosome were analyzed in three different cell types on tissue sections: CTBs in uterine wall (BP, invasive CTBs), multinucleated STBs that cover chorionic villi (ST), and mesenchymal cells in the central cores of chorionic villi (VC). At least 40 cells on each cell type were analyzed for each sample (n = 2–6). Progenitor CTBs and CTBs in the columns of anchoring villi, which were too tightly packed to resolve individual nuclei, were not scored. Fig 6 shows that less than 10% of STBs (ST) and mesenchymal cells (VC) were hyperdiploid, unlike a much higher rate of hyperdiploidy in CTBs in the uterine wall (BP, invasive CTBs). The most chromosomal gains were for chromosomes 19 (68%), 22 (66%), 15 (64%) and 14 (49%), while the least gains were seen for chromosomes 18 (12.4%), 9 (11%), 8 (8.9%) and 16 (7.3%). Two sample t-tests were performed (alpha = 0.025) to account for the multiple tests per sample. Significant differences were calculated between BP and the two other cell types. Significant differences were found on chromosomes 19, 22, 15, 14, 21, 13, and 8. Furthermore, the hyperdiploidy rates of the thirteen targeted chromosomes were compared between tissue invasive CTBs (*in vivo*) and invasive CTBs from explant (*in vitro*) as shown in Fig 7. The hyperdiploidy rate of each chromosome displayed a similar trend, with the highest copy number changes affecting chromosomes 15, 19 and 22; and the least copy number changes were observed for chromosomes 8, 9 and 16. Two sample t-tests were performed (alpha = 0.05). Significant differences between the means were found on chromosomes 6 and 9. For other chromosomes the null hypothesis that the true means of the *in vivo* and *in vitro* samples were the same could not be rejected.

## Discussion

The fetomaternal interface encompasses three main processes: establishment of immune tolerance, regulation of decidual invasion by CTBs, and uterine vascular remodeling by CTBs [26, 27]. This study has shown that human CTBs acquire widespread hyperdiploidy as they differentiate to invasive CTBs, suggesting that hyperdiploidy is an important component of normal placentation. This phenomenon is reminiscent of cancer cells as they undergo proliferation, migration, and metastasis [28]. However, the placenta has developed the ability to limit its invasiveness as pregnancy reaches term–the high heterogeneity of hyperdiploid CTBs may play an important role in this limiting mechanism.

Our findings show that the total number of chromosomes in the nucleus of each invasive CTB was very heterogeneous, ranging from 38 to 68 chromosomes. This raises the question: Why are these hyperdiploid CTBs not found in prenatal CVS diagnoses? First, CVS usually examines trophoblasts and mesenchyme cells in the floating villi, and both are characterized by their very low population of invasive CTBs. Second, CVS examines only proliferating cells, whereas, invasive CTBs have already exited the cell cycle [13]. Hyperdiploidy in invasive CTBs showed higher incidences for chromosome 19 (59%) and acrocentric chromosomes [chromosomes 13 (30%), 14 (34%), 15 (49%), 21 (52%), and 22 (56%)] than other chromosomes. Using next generation sequencing, analyzed trophoblasts of blastocysts showed that 40.9% of trophoblasts were aneuploid and 30.1% demonstrated mosaicism. Aneuploidy mostly affected chromosomes 22, 21, 16 and 15, while mosaicism was seen most frequently for chromosomes 21, 22, and 2 [29]. Nakhuda and colleagues [29] also suggested that chromosomes 19 and 22 were more susceptible to chromatid segregation errors. Invasive CTBs in our study also showed a similar trend except for chromosome 16. Chromatid segregation errors could be a mechanism to induce hyperdiploidy in CTBs during their differentiation phase when they enter the invasive pathway.

Like tumor cells, invasive CTBs use matrix metalloproteinases (MMPs) to digest extracellular matrix proteins in order to invade the epithelial basement membrane that separates these cells from the underlying uterine tissue [4]. In preeclamptic pregnancies, the part of the placenta that attaches to the uterine wall is severely affected by abnormal function of invasive CTBs [30, 31]. Copy number variant studies in human full-term placentas [32] showed that placentas from pregnancies with complications showed a lower number of somatic duplications compared to uncomplicated term placentas. Unpublished data on chromosome 18 and acrocentric chromosomes 13 and 21 revealed significantly fewer hyperdiploid invasive CTBs in preeclampsia pregnancies than in normal pregnancies: 11.7% (preeclampsia) vs. 44.7% (normal), p < 0.01 [manuscript in progress]. It supports the concept that abnormal CTB differentiation resulting in fewer hyperdiploid CTBs, precedes the onset of clinical symptoms of preeclampsia in pregnancies. Interestingly, CTBs isolated from placentas of preeclamptic pregnancies normalized their gene expression over 48 hours *in vitro* [33], suggesting that some aspects of the observed *in situ* aberrant differentiation of CTBs within the uterine wall, may be reversible. Therefore, the environmental effect (maternal-fetal interface stress) is important for CTB differentiation into the invasive pathway, which may induce chromosome segregation errors and in turn increases chromosome numbers.

Conversely, heterogeneous hyperdiploid CTBs could be the reason for the limited placental life span. The levels of proteins essential for DNA replication, chromosome condensation, repair or cell division are expected to be unbalanced in aneuploid cells, and this imbalance could promote the induction of DNA damage and replication defects [34, 35]. Therefore, the heterogeneity of chromosome composition in invasive CTBs may be one of the limiting factors of invasion and consequently, to the natural termination of pregnancy after nine months.

Furthermore, we have listed some known upregulated genes/proteins found in invasive human CTBs, its corresponding FISH probe location and the percentage of hyperdiploid CTBs, as shown in Table 2. Although Table 2 shows some correlation between the upregulated genes/proteins and hyperdiploidy rates, not all regions show the same trend. Our FISH results showed that most of the centromeric region probes (Fig 2, chr.1, 6, 8, 9, 10, 11, 12, 16, 17, and 18) have mean copy numbers similar to the control male sample except for chromosomes 7 and 10 (Fig 2, centromere probes with gain). The upregulated genes and proteins in invasive CTBs could be the reasons for an increase in the number of certain chromosomes and will need further studies to elucidate their functions in normal placentation and possibly tumorigenesis.

**Table 2:** A Summary of upregulated genes/proteins in placentas and current FISH study in invasive CTBs (iCTBs).

Upregulated genes/proteins*	Chromosome location	FISH probe in this study	Hyperdiploidy rate in iCTBs
*CTNNB1*	3p22.1	3q27.3	19.2%
Prolactin-like hormones	6p22.3	6cen, α-sat	3.5%
*EPHA7*	6q16.1		
*CSMD1*	8p23.2	8cen, α-sat	7.6%
Cathepsin B	8q23.1		
Cathepsin L	9q21.33	9cen, sat III	2.5%
*ADAM12*	10q26.2	10cen, α-sat	22.4%
*C2CD5*	12p12.1	12cen, α-sat	2.3%
serpin gene cluster	14q32.13	14q13.3	33.6%
*IGH*	14q32.33		
*hCG (CGB3 and CGB5)*	19q13.3	19q13.2	59.5%
*KIR*	19q13.4		
*MMP-9*	20q13.2	20q11	21.5%
*GnT-III*	22q13.1	22q11	56.0%

*The information for upregulated genes and proteins was taken from various studies [32, 36–42].

Currently, cell-free fetal DNA (cffDNA)–small fragments less than 200 bp in size–in maternal blood from placental trophoblasts are used for non-invasive prenatal testing (NIPT) [43, 44]. This study has shown that normal invasive CTBs are highly hyperdiploid which is supported by other findings of extensive somatic CNVs, rearrangements and mosaicism in trophoblasts [32]. These abnormalities may interfere with the reliable detection of the fetal CNV profile in NIPT using cffDNA. Therefore, any false positive cases in NIPT should be followed up using other prenatal testing methods [43].

In summary, human invasive CTBs acquire hyperdiploidy with a total chromosome number range from 47 to 53. Most copy number gains were predominantly seen on chromosomes 13, 14, 15, 19, 21 and 22. Such gains may relate to the upregulated genes responsible for inducing CTB invasion. These specific hyperdiploid CTBs could be one of the key factors to facilitate invasion in normal placental development.

## Supporting information

S1 Data (XLS)

S2 Data (XLSX)

S3 Data (XLSX)

S4 Data (XLS)
